# Effect of Glass-Composite Additives on the Properties of Cement-Based Products

**DOI:** 10.3390/ma18174031

**Published:** 2025-08-28

**Authors:** Wiktor Szewczenko, Galyna Kotsay

**Affiliations:** Faculty of Civil Engineering, Mechanics and Petrochemistry, Warsaw University of Technology, Łukasiewicza St. 17, 09-400 Płock, Poland; wiktor.szewczenko@pw.edu.pl

**Keywords:** water glass, waste glass, cement, alkali activity, composite, strength

## Abstract

In recent decades, replacing clinker in cement with mineral additives has become increasingly common, and the range of such additives continues to expand. An example is the growing number of cement types defined in European standards. Some of these standards allow the use of fine recycled concrete waste as an active additive in amounts up to 35%.. Finely ground waste glass, whose quantity steadily increases worldwide, can also be classified as an active additive. Due to its relatively high silica content, finely ground waste glass is a potential supplementary cementitious material. However, the high alkali content in glass limits its use in cementitious products to 5–10% of the binder mass. Considering that silicate binders (water glass) with high alkali activity are also used in construction and influence the hydration process of cement, it can be assumed that waste glass likewise has a significant impact on the properties of cement composites, particularly on hardening parameters and the development of mechanical strength. However, scientific literature lacks data on the synergistic effect of alkaline components of finely ground waste glass and water glass on the cement hydration process, its hardening, and the strength of cement products. Therefore, this study aimed to analyze the mechanism of the influence of a glass-based composite, consisting of waste glass additives and water glass, on the physicochemical and mechanical properties of cement composites.

## 1. Introduction

Most industrial silicate glasses, in addition to their primary component, silicon dioxide, also contain alkali oxides Na_2_O and K_2_O in amounts ranging from 4 to 14% [1,2,3,4,5]. The exact content of these oxides depends on the type of glass. The introduction of alkalis during glass production ensures the necessary technological properties of the glass melt (lower melting temperature, reduced viscosity of the molten glass) as well as appropriate properties of the finished products. Glass is a material that can be repeatedly recycled. Nevertheless, in Europe, the collection and recycling rate of glass waste is around 70–80% [6,7]. Recycled glass cullet finds wide application in construction, e.g., in the form of glass powder as a component of decorative tiles or plasters, as an abrasive, or in glass fibers used for thermal and acoustic insulation. However, the possibility of using waste glass cullet in cement-based products is limited, due to its high alkali content [8,9,10,11]. It is known that alkalis significantly affect the hydration process of cement. According to [12,13,14], sodium and potassium ions reduce the solubility of calcium hydroxide in cement paste and increase the solubility of clinker components such as alite and belite. Although they accelerate the dissolution of clinker components, leading to more hydration products and higher early strength, they also cause a more heterogeneous microstructure of the C-S-H phase and promote the formation of syngenite, which can result in reduced strength of the cement paste at later curing stages. In addition, alkalis from waste glass cullet migrate into the pore solution of concrete, which may cause alkali-silica reaction (ASR) in concrete [8,15,16,17,18].

The second type of glass with a high alkali content (approx. 8–10%) is sodium and potassium water glass. Despite its high alkali content, it shows different effects on the cement hydration process depending on the method of its introduction into the cement paste. According to [19], the addition of 5% water glass to the mixing water prolongs the hydration time of cement, while its introduction directly into the cement paste, conversely, accelerates hydration. A similar phenomenon is observed when using glass powder, although this effect is less pronounced [20]. This difference can be explained by the fact that the alkali component of water glass is in a dissolved form, whereas glass powder releases alkali cations only from the surface of the glass particles. It has been established that the amount of Na_2_O_eq_ extracted from the surface of glass particles is several times lower than the total alkali content in the bulk material. Therefore, considering the limitations on alkali content in cement, which should not exceed 0.6% Na_2_O_eq_ for low-alkali cement [21], when introducing water glass and powdered waste glass into cementitious composites, it is necessary to take their alkali activity under consideration [22].

In many previous studies, water glass has been used as an additive to regulate the setting time of cement, while waste glass has been applied as a non-clinker component in cementitious materials. However, the application of a glass-based composite combining water glass with finely ground waste glass in cement systems has not yet been thoroughly investigated. In the authors’ earlier work [23], it was shown that the inclusion of waste glass significantly enhances the polycondensation process of water glass and results in composites with high adhesive and cohesive properties. Moreover, the scientific literature lacks data on the synergistic effects of alkaline components derived from finely ground waste glass and water glass on cement hydration, hardening, and the mechanical strength of the resulting products.

Therefore, the aim of this study was to analyze the interaction mechanism between a glass-based composite—composed of waste glass and water glass—and the physicochemical and mechanical properties of cement-based materials.

## 2. Materials and Methods

The study used CEM II/A-LL 42.5R-NA cement produced by Cement Ożarów S.A. (Ożarów, Poland) [24], sodium and potassium water glasses produced by Chemical Plant “Rudniki” S.A. (Rudniki, Poland) [25], and waste glass provided by REWA (Koluszki, Poland) [26] in the form of glass powder with a particle size below 0.063 mm. The chemical compositions and properties of the materials are presented in Table 1. The silicate modulus and density of the sodium and potassium water glasses were determined using the methods described in standards [27,28]. The density and Blaine specific surface area of the cement and waste glass were determined in accordance with the methods described in standards [29,30].

Waste glass and water glass were added to the cement in amounts of 5% and 10% by mass, exceeding 100% of the base cement mass. A total of 11 mixtures were prepared: one reference mixture, six mixtures containing a single additive (either water glass or waste glass), and four mixtures with a complex additive combining waste glass with either sodium or potassium water glass.

To assess the alkaline activity of the individual components and compositions, a flame photometer FP902 (PG Instruments Limited, Alma Park, Wibtoft, Leicestershire, UK) with an accuracy of ±0.5% was used. Distilled water served as the extractant. The alkaline activity of the glass polymer compositions was determined based on a series of six specimens, and the results are presented in units of ppm/dm^2^.

The effect of waste glass and water glass on cement hydration was evaluated using a Calmetrix I-Cal 2000 HPC isothermal calorimeter (Boston, MA, USA). Cement pastes were prepared with a liquid-to-binder ratio of 0.5. The water glass was dissolved in the mixing water, while the waste glass powder was blended with the cement before mixing. The mixtures were homogenized for 1 min using a laboratory mixer. The heat release rate was continuously recorded every 30 s over 48 h under isothermal conditions at 20 °C. The rheological measurements (dynamic viscosity) were conducted using the IKA ROTAVICS me-vi (Staufen im Breisgau, Germany). The pastes of water glass and waste glass were prepared in a mixer. The mixing time was 3 min. The measurement of rheological parameters was conducted 5 min after mixing the components.

The flexural and compressive strengths of mortars made with glass–cement composites were determined after 2 and 28 days of cement hydration, according to [31]. The adhesive strength of the hardened mortars on substrates was determined according to [32]. As the adhesive paste, a composition containing water glass and powdered waste was used in the following proportions: SWG:WG in a ratio of 1:2 with a viscosity of 21 Pa·s and PWG:WG in a ratio of 1:1 with a viscosity of 23 Pa·s. This difference results from the fact that the paste with PWG is more viscous and difficult to apply to the surface of the cement composite. The layer of adhesive paste was applied to the fracture surface of the beam specimens after the tensile bending test, after which the specimens were joined, and the excess paste was removed. The bonded specimens were stored vertically under room conditions for 14 days. Subsequently, the bonded beams were subjected to a bending test to determine their tensile strength, with the breaking force applied along the bond line.

## 3. Results and Discussion

The alkali activity (AA) of a solid should be understood as the amount of alkaline components extracted from the material’s surface within a short period (so-called free alkalis); in other words, only the kinetic component of the extraction process is considered, while the diffusion component is disregarded. Table 2 shows the alkali activity of the materials used: cement, waste glass, and water glass.

In the chemical composition of the cement used, the content of active potassium oxide is approximately eight times higher than that of sodium oxide, whereas in waste glass, the situation is reversed—the content of active sodium oxide is about one and a half times higher than that of potassium oxide. Compared to waste glass, water glass is an aqueous solution of sodium or potassium silicates and their hydrolysis products; therefore, the content of active alkalis corresponds to their amount in its chemical composition [32,33,34].

In cement chemistry, the total alkali content is usually expressed as the sodium oxide equivalent, calculated according to the formula Na_2_O_eq_ = Na_2_O + 0.658·K_2_O. If the alkali content is expressed in terms of sodium oxide equivalent (Table 2), the highest amount of active alkalis (Na_2_O_eq_) is introduced by sodium and potassium water glass, followed by waste glass, with the lowest amount found in cement.

In order to determine the effect of different types of high-alkali glass on the alkali activity of the final cement composite, waste glass and water glass were added to the cement at levels of 5% and 10% by mass of cement, according to the results of our previous studies [35,36]. The composition of the composites and the alkali activity of the cement-based composites are presented in Table 3. Based on the alkali activity of the individual components (Table 2), the content of active alkalis (Na_2_O_eq_) in the tested cement composites was calculated using an additive method. Additionally, the alkali activity of the composites was measured after hydration of the cement composite following 28 days of curing.

The results presented in Table 3 clearly indicate that the alkali activity (AA) of the cement composites depends significantly not only on the type and amount of the additive used but also on the combined interaction of different alkaline additives. It is worth emphasizing that the relationship between the AA of the composite substrate and the AA of the hydrated composite product is not linear.

Calculations of alkali activity showed that the greatest deviations from the permissible value (0.6%) occur when 10% of sodium or potassium water glasses were added. In contrast, the introduction of finely ground waste glass in the same amount does not exceed the permissible limits. This difference can be explained by the fact that water glass completely dissolves in the mixing water, while glass powder releases only surface alkali cations.

For the hydrated composites with the addition of sodium water glass (SWG), a nonlinear character of changes in alkali activity was observed. The addition of 5% SWG resulted in a decrease in the alkali activity (AA) of the adhesive paste compared to the reference sample, despite the increased alkali content in the initial materials. This phenomenon is related to the polyalkali effect described in [36]. However, increasing the SWG content to 10% caused a significant increase in alkali activity (AA), which indicates exceeding the threshold capacity for alkali binding and an increase in the amount of free Na^+^ ions on the composite surface.

The addition of potassium water glass (PWG) increased the alkali activity of the hydrated cement composites more significantly than sodium water glass (SWG), especially at a 10% content, reaching a maximum value of 4.40 ppm/dm^2^. The increased content of potassium ions was associated with their higher concentration compared to the sodium ions present in the cement. Therefore, the introduction of potassium water glass does not reduce the alkali activity to the same extent as sodium water glass applied at a 5% content.

The polyalkali effect was also observed in the case of using sodium–calcium waste glass (WG). Both the independent use of this additive and, in particular, its combination with potassium water glass in the PWG10 + WG10 system resulted in a reduction in the alkali activity (AA) compared to the reference sample. The AA value of the hydrated mortar in this case was the lowest among all the systems tested.

The obtained results indicate that appropriately selected additives, especially waste glass in combination with other alkaline additives, can effectively reduce the AA of composites and thus contribute to improving their durability.

**Figure 1:**
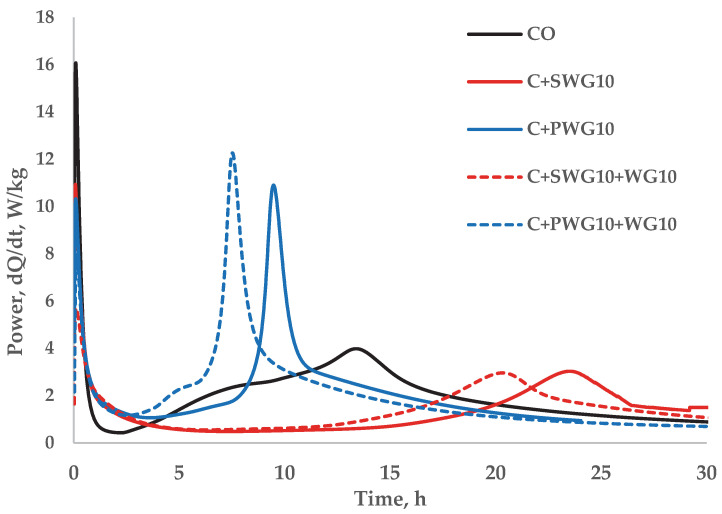
The calorimetric curves of cement hydration with glass additives.

It is well known that alkalis have a significant impact on the hydration of the binder and consequently on the strength of the hydration products. Synergistic interactions of waste glass and sodium or potassium water glass are also evident during cement hydration. Figure 1 presents the calorimetric study of the hydration process of cement composites using a 10% addition of sodium and potassium water glass as well as the composite of water glass and waste glass.

Based on the calorimetric analysis (Table 4), it was found that the addition of waste glass (WG) slightly increases the rate of cement hydration both in the pre-induction and post-induction periods, while sodium water glass (SWG) clearly slows down the hydration process and reduces the released heat by half, which may result from the formation of an ionic barrier around the cement grains. In turn, potassium water glass (PWG) shows moderate retardation in the initial phase but significantly accelerates the hydration reactions in the post-induction period. The composites (SWG + WG and PWG + WG) exhibit a synergistic effect: in the case of SWG + WG, hydration is strongly suppressed, whereas PWG + WG significantly intensifies hydration in the later stage, resulting in the highest released heat and reaction intensity during this phase.

**Figure 2:**
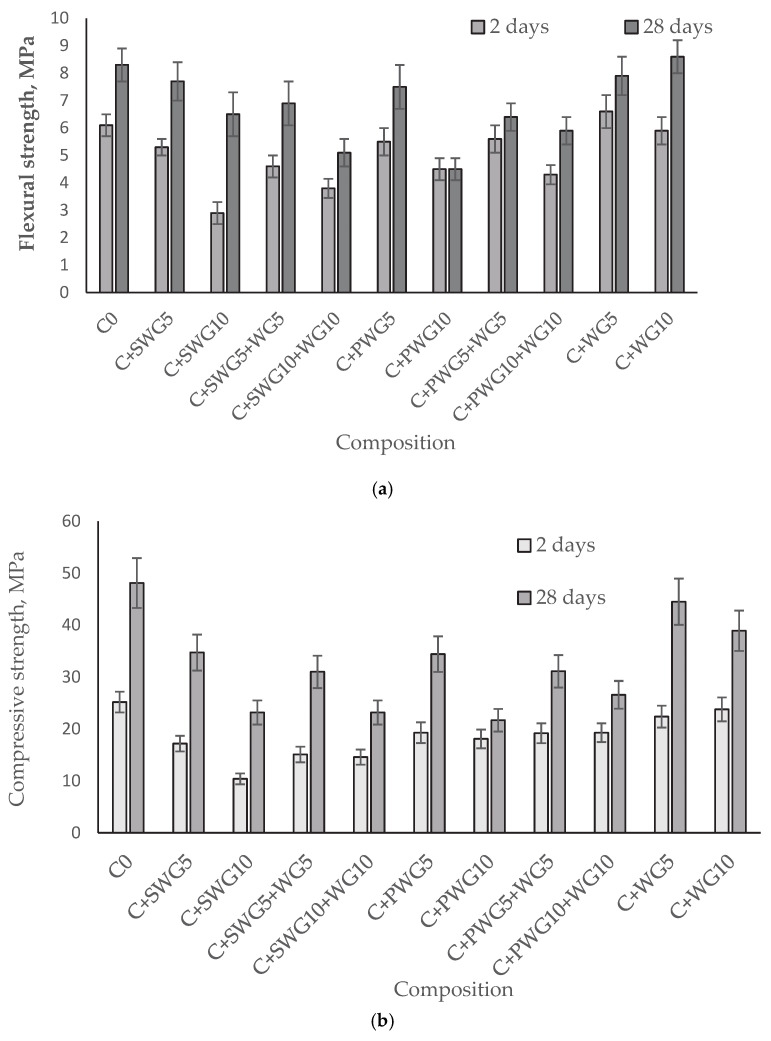
Mechanical strength of cement composites with glass additives: (**a**) flexural strength, (**b**) compressive strength.

Considering that the strength of hydrated cement reflects its structural (cohesive) strength, the mechanical strength of all glass–cement composites was determined after 2 and 28 days of cement hydration in accordance with the European standard PN-EN 196-1 [31] (Figure 2).

First of all, it should be noted that CEM II A/LL 42.5R-NA meets the requirements of the EN 197-1 standard [37] for early and standard compressive strength. Based on the conducted tests, it was found that the introduction of sodium water glass (SWG) and potassium water glass (PWG) into cement mortars negatively affects their mechanical properties, both in terms of flexural and compressive strength. In the case of mortars modified with a 5% addition of SWG (C + SWG5), a decrease in compressive strength after 28 days was observed, from 48.10 MPa (reference value) to 34.70 MPa, while with a 10% SWG addition (C + SWG10), this decrease was even more pronounced, reaching 23.20 MPa. These observations indicate that sodium water glass, particularly in higher amounts, negatively influences the development of the strength structure of the cement paste.

A similar trend was observed in the case of potassium water glass (PWG). The addition of 5% PWG (C + PWG5) reduced the compressive strength after 28 days to 34.40 MPa, while 10% PWG (C + PWG10) caused a significant decrease to 21.70 MPa. Although both forms of water glass reduce the mechanical parameters of mortars, potassium water glass shows a slightly milder effect than its sodium counterpart, especially in the early curing period (2 days).

In contrast, the addition of waste glass (WG) in amounts of 5% and 10% does not cause a significant deterioration in mechanical properties. For systems containing only WG (C + WG5 and C + WG10), compressive strengths after 28 days reached 44.50 MPa and 38.90 MPa, respectively, which indicates a relatively favorable influence of this additive. Additionally, the flexural strength of the C + WG10 sample (8.60 MPa) was higher than that of the reference sample (8.30 MPa), which may suggest a beneficial effect in terms of pore microfilling and the potential pozzolanic reactivity of the waste glass.

In the case of combinations of activators (SWG or PWG) with waste glass (e.g., C + SWG5 + WG5, C + PWG10 + WG10), a partial compensation of the negative effect of water glass by the addition of WG was observed. However, despite the improvement compared to systems containing only SWG or PWG, the strengths still did not reach the level of the reference sample.

Since water glass affects the adhesive strength of cement composites [19], an attempt was made to investigate the influence of the chemical composition of the composites on their adhesive strength with respect to cement composites, namely water glass and waste glass. As the adhesive paste, a composition containing water glass and powdered waste was used in the following proportions: SWG:WG in a ratio of 1:2 with a viscosity of 21 Pa·s and PWG:WG in a ratio of 1:1 with a viscosity of 23 Pa·s. This difference results from the fact that the paste with PWG is more viscous and difficult to apply to the surface of the cement composite. The layer of adhesive paste was applied to the fracture surface of the beam specimens after the tensile bending test, after which the specimens were joined. Subsequently, the bonded beams were subjected to a bending test to determine their tensile strength. The strength results and the failure modes are presented in Table 5.

**Figure 3:**
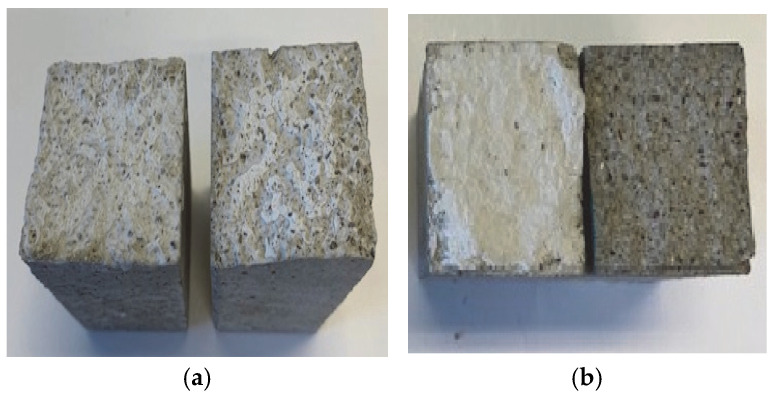
Glued beam samples after bending failure: failure surface with adhesive paste residues on both surfaces, f_adh_ > f_coh_; (**a**) failure surface with adhesive paste residues on one of the sample surfaces, f_adh_ < f_coh_ (**b**).

A visual assessment of the fracture zone allowed us to clearly determine whether the obtained strength values characterized the cohesive (structural) strength of the adhesive paste or its adhesion strength to the surface of the cement composite. Figure 3 shows the fracture surface of the bonded beam specimens after failure. The photograph clearly indicates that the presence of adhesive paste residues on both halves demonstrates that the obtained strength values represent the cohesive strength of the adhesive paste, while residues on only one half indicate that the measured strength corresponds to the adhesion of the paste to the fracture surface of the beam. This interpretation is reflected in Table 5, where f_adh_ characterizes the adhesive strength of the paste, and f_coh_ refers to its cohesive strength.

The examination and visual analysis of the damage zone showed that in most cases, the fracture occurred along the hardened adhesive paste. This provides a basis for interpreting the bending strength as the cohesive strength of the adhesive composition. Considering the relatively uneven damage surface, the confidence interval for processing the results of determining the cohesive strength of the adhesive compositions (pastes) was assumed to be ±20%. Thus, based on the results presented in Table 5, the cohesive strength of the adhesive composition based on SWG is approximately 3.2 MPa, and based on PWG, it is 3.0 MPa, while the adhesive strength is 4.7 and 1.6 MPa, respectively. From this, it can be concluded that the cohesive strength of the adhesive paste for different types of liquid glass differs only slightly. However, the adhesive strength for sodium water glass is more than 30% higher than its cohesive strength, whereas for potassium water glass, it is 47% lower. This difference can be explained by the different composition of sodium and potassium adhesive pastes.

Moreover, visual analysis of the fracture surfaces revealed that air pores with a diameter of 0.2–0.5 mm were not filled with adhesive paste, which indicates a high viscosity and high surface tension of the adhesive substance. Therefore, the mechanism of adhesion of the paste to the surface of the hardened cement composite cannot be explained from the perspective of mechanical adhesion theory but is rather a consequence of the formation of chemical bonds between them.

To test this assumption, adhesion strength tests were carried out on the bond between the surface of the cement composite and the adhesive paste consisting of water glass and waste glass, with and without preliminary dealkalization of the fracture surface of the cement composite. In this case, it was assumed that the dealkalization process is one of the methods of modifying the surface of the cement stone by extracting alkali cations according to reaction (1):

R^+^ (cement composite) + H-OH (extractant) ↔ ROH (solution) + H^+^ (cement composite) (1)

where R^+^ = Na^+^, K^+^.

Chemical reaction (1) is a typical ion-exchange reaction, in which the alkali cations of the cement stone in an aqueous environment (the extractant) are replaced by hydrogen cations from water. In this case, the ionic bond Si-O^−^ + R, with a bond energy of 600–1100 kJ·mol^−1^, is replaced by a hydrogen bond Si-O^−^ + H, with a bond energy of 1–25 kJ·mol^−1^ [23]. Such a replacement increases the reactivity of the cement stone surface with respect to, for example, adhesive substances, thereby enhancing the adhesion strength at the phase boundary. The dealkalization process was carried out by immersing the fractured surface in distilled water at 90 °C for 20 s. After drying, the divided surface was coated with the adhesive paste, and then the halves were bonded together and kept in a vertical position for 14 days. Afterwards, the bonded specimens were subjected to a tensile bending test (Rten), as shown in Table 6.

First of all, it should be noted that in all cases, the tensile strength of the bonded specimens is 20–40% lower than that of the unbonded (standard) beam (8.3 MPa). An increase in the content of powdered glass in the adhesive paste, in all cases and regardless of the type of liquid glass, leads to a reduction in the cohesive strength of the adhesive pastes, both with and without substrate dealkalization. For example, for SWG, this decrease ranges from 2.1 to 5.3% with a twofold increase in the amount of WG, and for PWG, it ranges from 13.1 to 27.6%. If at an SWG:WG ratio of 1:1 and 1:2 the adhesion strength was higher than the cohesive strength, then at a ratio of 1:3, conversely, the adhesion strength becomes lower than the cohesive strength for both SWG and PWG. This may indicate that with a higher amount of WG, its specific surface area increases, which requires more water glass to form the internal structure of the adhesive paste. As a result, the cohesive strength of the paste increases while its adhesive capacity decreases, thus reducing the adhesion strength at the substrate–paste interface.

The use of water glasses without the addition of glass powder as an adhesive shows a threefold to fivefold decrease in the cohesive strength of the pastes compared to pastes containing glass powder. This may serve as confirmation of the previously expressed assumption that the increase in cohesive and adhesive strength is due to the participation of additional alkaline cations from the glass in forming additional ionic bonds, both within the adhesive substance and in its bond with the cement composite, as a result of secondary ion exchange between the adhesive paste and the cement composite. In view of the above, the entire process of forming the bonding zone can be presented as follows:

K^+^(substrate) + H-OH(extractant) ↔ KOH(solution) + H^+^(substrate) (2)

H^+^(substrate)) + Na^+^(class. paste) ↔ Na^+^(substrate) + H^+^(class. paste) (3)

Thus, due to the two-stage ion exchange on the surface of the substrate, a weak hydrogen bond Si-O-H was formed as a result of dealkalinization, which was replaced by an ionic bond Si-O-Na, which is more than 20 times larger and ensures high adhesive strength of the adhesive paste.

## 4. Conclusions

Research has shown that adding waste glass together with sodium and potassium water glass to cement composites has a significant impact on their physical, chemical, and mechanical properties. Finely ground waste glass demonstrated potential as a cement additive without significantly reducing mortar strength. However, the use of sodium and potassium water glasses, especially at higher dosages (10%), resulted in a considerable decrease in compressive and flexural strength, primarily due to increased alkali activity and disruption of the cement hydration process. Calorimetric analyses revealed that aqueous sodium water glass tends to slow down hydration and reduce the heat of reaction, while aqueous potassium water glass can moderately accelerate hydration at later stages.

The studies clearly showed that the use of glass-composite additives, combining waste glass and water glass, significantly affects the alkali activity of cementitious materials. The results confirmed that water glass, particularly in a 10% dosage, increases the alkali activity of the hydrated composite due to its high solubility and rapid release of alkali cations. In contrast, finely ground waste glass showed a stabilizing effect on alkali activity by limiting the amount of free alkalis available in the pore solution.

The most noteworthy finding was obtained for the composite glass system containing potassium water glass together with waste glass (PWG10 + WG10), where the alkali activity of the hydrated composite reached the lowest value among all tested variants. This confirms the presence of a synergistic polyalkali effect, in which the combination of multiple alkali sources, introduced in controlled proportions, leads to more effective binding and immobilization of alkalis in secondary hydration products. These results highlight the significant role of alkali-related factors in determining the long-term durability of cement-based materials. Therefore, controlling and optimizing the alkali content in glass-based additives will be an important direction for future research.

The combination of waste glass with water glass partially compensated for the negative effects of water glass on mechanical properties but did not restore the strength to reference levels. In addition, the adhesion of pastes based on water glass and waste glass depends on their component ratios, and dealkalization of the cement surface further improved bonding performance. These results suggest that a carefully balanced use of waste glass with limited amounts of water glass can improve the sustainability of cement composites while maintaining satisfactory mechanical properties.

## 5. Patent

PL237507B1 Method of determining the alkaline activity of cement products.

## Figures and Tables

**Table 1:** Characteristics of sodium and potassium water glasses and fine-ground glass waste.

Materials	Oxides (wt %)						Silicate Modulus	Density, g/cm^3^	Specific Surface, m^2^/g
	SiO_2_	Na_2_O	K_2_O	R_2_O_3_	CaO + MgO	H_2_O			
Sodium water glass (SWG)	26.38	8.02	-	-	-	65.6	3.4	1.45	-
Potassium water glass (PWG)	21.41	-	7.59	-	-	71.0	4.4	1.25	-
Waste glass (WG)	72.00	10.50	4.50	1.00	12.00	-	-	2.43	~0.4
Cement (C)	21.30	0.51 *		9.52	68.67	-	-	3.06	0.54

* Na_2_O_eq_ = Na_2_O + 0.658·K_2_O.

**Table 2:** Alkali activity of cement, waste glass, and water glass.

Material	Amount of Extracted,%,%		Amount Na_2_O_eq_, %
	Na_2_O	K_2_O	
Cement	0.05	0.39	0.31
Waste glass	0.41	0.27	0.59
Sodium water glass	8.02	-	8.02
Potassium water glass	-	7.59	4.99

**Table 3:** Alkali content of initial materials and cement composites after hydration.

N	Composite Symbol	Composition of the Cement Composite, %				Content of Active Alkalis in the Composite, Na_2_O_eq,_ %	Alkali Activity of the Hydrated Cement Composite, ppm/dm^2^
		Cement	SWG	PWG	WG		
1	C0	100	-	-	-	0.31	2.20
2	C + SWG5	100	5.0	-	-	0.71	1.40
3	C + SWG10	100	10.0	-	-	1.11	3.50
4	C + SWG5 + WG5	100	5.0	-	5.0	0.74	2.10
5	C + SWG10 + WG10	100	10.0	-	10.0	1.17	3.60
6	C + PWG5	100	-	5.0	-	0.56	2.40
7	C + PWG10	100	-	10.0	-	0.81	4.40
8	C + PWG5 + WG5	100	-	5.0	5.0	0.59	3.00
9	C + PWG10 + WG10	100	-	10.0	10.0	0.87	1.00
10	C + WG5	100	-	-	5.0	0.34	2.00
11	C + WG10	100	-	-	10.0	0.37	1.50

**Table 4:** Characteristic calorimetric of cement hydration with glass additives.

Composition	The Heat of Hardening After 24 h, kj/kg	Maximum Rate Evolution of Heat in the Pre-Induction Stage of Hydration, W/kg	Maximum Rate Evolution of Heat in the Post-Induction Stage of Hydration, W/kg
C0	190.9	16.1	4.0
C + WG10	196.0	16.5	4.3
C + SWG10	106.1	10.9	3.0
C + PWG10	194.7	10.3	10.9
C + SWG10 + WG10	121.6	5.7	3.0
C + PWG10 + WG10	204.1	8.0	12.3

**Table 5:** Tensile strength in bending of adhesive paste.

Water-Glass–Waste-Glass Adhesive Composite Paste	Flexural Tensile Strength of Cement Mortar Bonded with Adhesive Paste, Mpa										
	C0	C + SWG5	C + SWG10	C + SWG5 + WG5	C + SWG10 + WG10	C + PWG5	C + PWG10	C + PWG5 + WG5	C + PWG10 + WG10	C + WG5	C + WG10
**SWG:WG/1:2**	4.0	5.0	2.2	4.0	2.7	4.6	4.8	3.0	3.6	5.3	5.6
Failure mode	f_coh_	f_adh_	f_coh_	f_adh_	f_coh_	f_coh_	f_coh_	f_coh_	f_adh_	f_adh_	f_adh_
**PWG:WG/1:1**	5.2	3.2	1.7	3.4	1.5	2.7	3.4	2.5	1.2	5.3	1.7
Failure mode	f_coh_	f_adh_	f_coh_	f_coh_	f_adh_	f_coh_	f_coh_	f_coh_	f_adh_	f_coh_	f_adh_

**Table 6:** Bonding strength depending on the surface condition and composition of adhesive pastes.

Composition of Adhesive Paste	Viscosity, Pa·s	Without Dealkalization		With Dealkalization	
		R_ten_, MPa	Type of Strength	R_ten_, MPa	Type of Strength
SWG:WG/1:1	0.67	4.8	cohesive	5.7	cohesive
SWG:WG/1:2	21	4.7	cohesive	5.4	cohesive
SWG:WG/1:3	>50	0.2	adhesive	1.6	adhesive
PWG:WG/1:1	22.72	4.6	cohesive	5.8	cohesive
PWG:WG/1:2	>50	4.0	cohesive	4.2	cohesive
PWG:WG/1:3	>>50	0.1	adhesive	0.4	adhesive
SWG-100%	0.09	1.1	cohesive	1.7	cohesive
PWG-100%	0.10	2.6	cohesive	1.3	cohesive

## Data Availability

The original contributions presented in the study are included in the article. Further inquiries can be directed to the corresponding author.
